# Characterization of CRISPR-Cas systems in the *Haemophilus* genus CRISPR-Cas in *Haemophilus* spp.

**DOI:** 10.1590/1678-4685-GMB-2025-0166

**Published:** 2026-03-16

**Authors:** Ying Huang, Xinchao Yi, Xue Yang, Chao Li, Yuan Li, Zufeng Ye, Jun He

**Affiliations:** 1 The Affiliated Nanhua Hospital, Department of Clinical Laboratory, Hengyang Medical School, University of South China, Hengyang, China.; 2 The Second Affiliated Hospital, Department of Clinical Laboratory, Hengyang Medical School, University of South China, Hengyang, China.

**Keywords:** Haemophilus, CRISPR-Cas system, virulence gene, genome evolution, phage

## Abstract

Clustered Regularly Interspaced Short Palindromic Repeats (CRISPR) and CRISPR-associated (Cas) system constitutes a crucial adaptive defense mechanism in prokaryotes against foreign genetic elements. Although CRISPR-Cas systems have been characterized in numerous bacteria, the architecture and function of these systems in the *Haemophilus* genus remain poorly understood. This study aims to analyze CRISPR-Cas systems in 138*Haemophilus*strains and investigate their function, particularly in relation to virulence factors. Results revealed that CRISPR-Cas systems were identified in 31.88% of the *Haemophilus*strains. Subtype I-C was the most prevalent, followed by subtypes II-C and III-A. Repeat sequences and the*cas1*gene were highly conserved within the same subtype. 29.62% of spacer sequences exhibited homology to plasmids or bacteriophages. phiMHaA1 was an important target of the CRISPR-Cas system in *Haemophilus*genus. The protospacer adjacent motif sequences (PAM) were determined to be 5′-TTC-3′ for subtype I-C and 5′-TTT-3′ for subtype II-C. Comparative analysis of virulence genes showed that CRISPR-positive strains carried more *ompP2* than CRISPR-negative strains, while the distribution of *hmw2C* and *hmw1C* exhibited an opposite trend. These findings provide novel insights into the diversity and function of CRISPR-Cas systems in*Haemophilus*genus and propose potential strategies for attenuating the impact of*Haemophilus*virulence factors.

## Introduction

*Haemophilus* spp., a member of the *Pasteurellaceae* family, is commonly found in the human respiratory tract (Dewhirst et al., 1992). The *Haemophilus* genus is composed of diverse species, including *Haemophilus influenzae*, *Haemophilus parainfluenzae*, and *Haemophilus ducreyi* (Albritton, 1982). *Haemophilus spp.* can cause respiratory infections and invasive diseases, ranging from community-acquired pneumonia to life-threatening meningitis (Wen et al., 2020). The virulence factors of *Haemophilus* spp. include capsule, pili, adhesins, and IgA protease (Kostyanev and Sechanova, 2012). Adhesins are a large class of proteins including HWM1/HWM2, Hia/Hsf, Hap, and outer membrane proteins, which regulate bacterial adhesion and colonization. These proteins are respectively encoded by the genes *hmw1ABC/hmw2ABC, hia/hsf, hap, omp5, and omp2* (Xiao et al., 2023). The pili, encoded by the *hifABCDE gene,* are involved in bacterial adhesion, colonization, and biofilm formation (Mokrzan et al., 2018). IgA proteases facilitate bacterial colonization and tissue invasion (Murphy et al., 2015). The capsule allows bacteria to evade host immunity, which is closely associated with *bexABCD* genes (Xiao *et al.*, 2023). These virulence genes are usually found in mobile gene elements (MGEs) and can be transmitted between strains by horizontal gene transfer (HGT) mediated by these mobile gene elements (Hiltke et al., 2003).

As a result of sustained selective pressure, bacteria have evolved numerous defense mechanisms. The clustered regularly interspaced short palindromic repeats (CRISPR)-CRISPR-associated proteins (Cas) system is one of the prokaryotic adaptive immunity systems against foreign DNA from phages, plasmids, and other MGEs. The CRISPR-Cas system mainly consists of three components: (i) CRISPR arrays that contain repeat sequences and spacers; (ii) the *cas* genes, encoding proteins critical for the adaptive immune response; (iii) the leader sequence (Wang et al., 2022). Additionally, the protospacer adjacent motif (PAM) plays a significant role in the precise recognition of target sequences (Shah et al., 2013). Currently, the CRISPR-Cas system has been found in diverse bacterial species, and it exhibits considerable diversity across different species. Based on the updated classification, CRISPR-Cas systems are divided into two classes, six types, and 33 subtypes (Makarova et al., 2020).

In recent years, the characterization of the CRISPR-Cas system has been reported for numerous bacteria, such as *Enterococcus*, *Klebsiella*, and *Bifidobacterium breve* (Han et al., 2022; Tao et al., 2023; Xu et al., 2024). Moreover, increasing evidence has shown that the CRISPR-Cas system may influence bacterial virulence (Louwen et al., 2014). The type I CRISPR-Cas system has been found to target the LasR mRNA in *Pseudomonas aeruginosa* to reduce the pro-inflammatory response of the host. This mechanism helps *Pseudomonas aeruginosa* evade host immunity (Li et al., 2016). There is a negative association between the CRISPR-Cas system and enterococcal surface protein (*esp*) gene in *Enterococcus* (Tao *et al.*, 2022). In another study, the *Klebsiella pneumoniae* isolates with the CRISPR-Cas system are more likely to produce biofilms and carry more virulence genes (Kadkhoda et al., 2025). However, there is limited information on the CRISPR-Cas system in *Haemophilus* spp. and its relationship with virulence genes.

Therefore, we elaborated the occurrence and characteristics of CRISPR-Cas systems in 138 *Haemophilus* strains, investigated the diversity of virulence genes of selected strains, and further evaluated the association between the CRISPR-Cas system and virulence genes in *Haemophilus* spp.

## Material and Methods

### Data collection and CRISPR-Cas system identification

As of November 2023, a total of 138 non-duplicated *Haemophilus* genomes were downloaded from NCBI GenBank (https://www.ncbi.nlm.nih.gov/genbank/), including 52 complete genomes and 86 draft genomes, all meeting “completion level” or “high-quality draft level” standards (Contig N50 value >70 kb) (Table S1). This significantly reduced misidentifications caused by genomic fragmentation. The CRISPRCasFinder (https://crisprcas.i2bc.paris-saclay.fr/CrisprCasFinder/Index) was used to identify CRISPR loci (Couvin et al., 2018). Only CRISPR arrays with evidence level 3 or 4 were retained for subsequent studies. The classification of identified CRISPR-Cas systems was performed by CRISPRCasTyper (https://typer.crispr.dk/#/submit) using default parameters (Russel et al., 2020). The CRISPRone **(** https://omics.informatics.indiana.edu/CRISPRone **)** was used to visualize the structure of CRISPR arrays.

### Characterization of repeat sequences and phylogenetic analysis

Repeat sequences, spacer sequences, and Cas sequences were downloaded from the CRISPRCasFinder server. Repeat sequences were submitted to the RNAfold web server (http://rna.tbi.univie.ac.at/cgi-bin/RNAWebSuite/RNAfold.cgi) to predict secondary structures and minimum free energy (MFE). The ClustalW algorithm was used for multiple alignments of cas1 genes and repeat sequences. Neighbor-joining trees were generated using MEGA-X 11.0 (Han et al., 2022). The phylogenetic trees were optimized using the iTOL web server (https://itol.embl.de/) (Letunic and Bork, 2021). 

### Spacer analysis and PAM prediction

All spacer sequences were submitted to the Clustal Omega server (https://www.ebi.ac.uk/jdispatcher/msa/clustalo) to create the percent identity matrix. Strains with highly similar spacer sequences were selected to visualize the arrangement of the spacer sequences using the Excel Macro. The protospacer was predicted using the CRISPRTarget server (http://crispr.otago.ac.nz/CRISPRTarget/crispr_analysis.html) limited to the RefSeq-Plasmid, GenBank-Phage, and PHAST databases (Biswas et al., 2013). The sequence with ≥85% identity to the spacer sequence was regarded as a strong protospacer sequence. The heatmaps for spacer matches were subsequently completed using the Origin software. The eight nucleotide flanking regions on the 5′ and 3′ ends of the protospacers were aligned, and the predicted PAM sequences were visualized using the WebLogo server (https://weblogo.berkeley.edu/logo.cgi) (Crooks et al., 2004).

### Identification of virulence genes

Virulence genes were identified using the VFDB database (http://www.mgc.ac.cn/VFs/) with the parameters (E-value< 0.00001 and identity ≥ 85%) (Liu et al., 2022). The investigated virulence genes included *hmw1A, hmw1B, hmw1C, hmw2A, hmw2B, hmw2C, hia, hsf, hap, omp5, omp2, hifA, hifB, hifC, hifD, hifE, bexA, bexB, bexC, bexD, and iga*.

### Statistical analysis

The SPSS 27.0 software was used for all statistical analyses. The chi-square test and Fisher’s exact test were utilized to analyze the relationship between the CRISPR-Cas system and virulence genes in *Haemophilus* genus. P < 0.05 was considered statistically significant.

## Results

###  Prevalence and structure of CRISPR-Cas systems in *Haemophilus*

**Figure 1 -:**
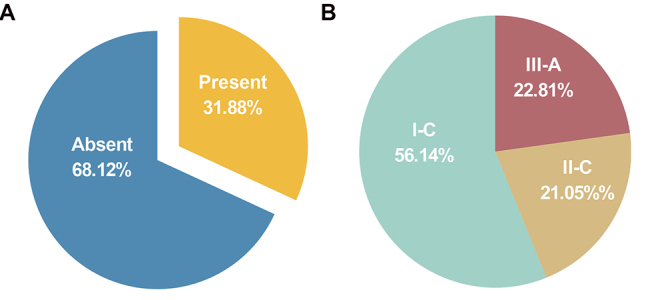
Occurrence of CRISPR system in *Haemophilus* strains. (a) Presence of CRISPR-Cas system. (b) Classification of CRSIPR-Cas systems.

The collection of 138 *Haemophilus* strains was investigated for the presence of CRISPR-Cas systems. The results showed that 57 CRISPR-Cas systems were identified, and these CRISPR-Cas systems were present in 31.88% (44/138) of the *Haemophilus* strains (Figure 1a). The 57 CRISPR-Cas systems spanned multiple *Haemophilus* species, including 20 *Haemophilus haemolyticus*, 20 *Haemophilus parainfluenzae*, 4 *Haemophilus influenzae*, 4 *Haemophilus* seminalis, 4 *Haemophilus sputorum*, and 5 unclassified *Haemophilus* strains (Table S2). Based on CRISPRCasTyper predictions, three types of CRISPR-Cas systems occurred in the genus *Haemophilus*. subtype I-C (56.14%) was the most common CRISPR-Cas system in *Haemophilus*, followed by subtype III-A (22.81%) and subtype II-C (21.05%) (Figure 1b). Most of the strains had only one type of CRISPR-Cas system, while a few strains carried multiple subtypes. Strain M1C152_1 and strain CCUG_13788 contained both subtype I-C and II-C systems, while strains 65151_B_Hi-4 and M1C142_1 carried both subtype I-C and III-A systems (Table S2).

**Figure 2 -:**
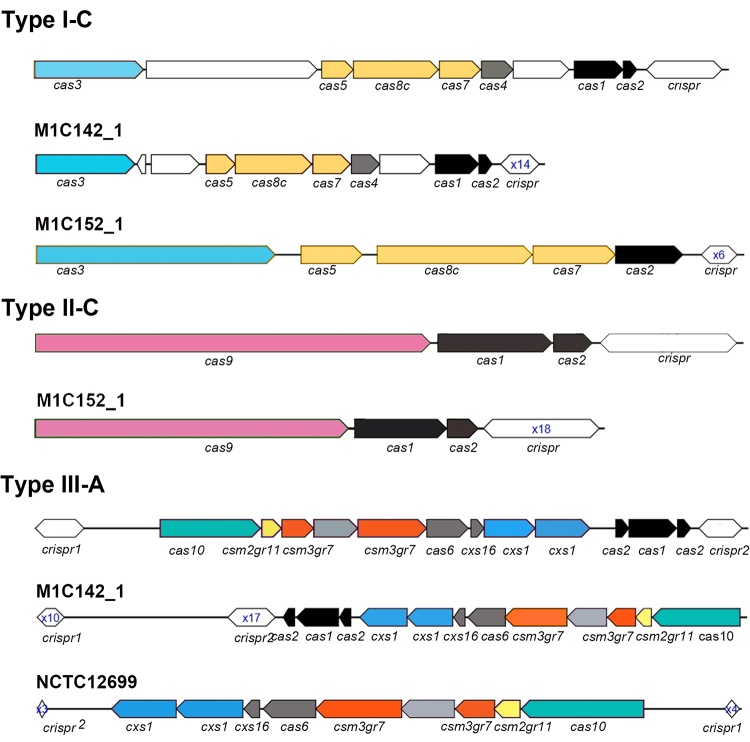
Schematic diagram of CRISPR locus in *Haemophilus* strains. The *cas* genes are represented with different colours and shapes. The CRISPR arrays are shown by double-headed arrow. The number within the double-headed arrow indicates the quantity of repeat sequences**.**

Further analysis revealed that CRISPR-Cas systems of the same type shared a set of similar structures. Type I-C shared a set of identical cas gene clusters (*cas3-cas5-cas8c-cas7-cas4-cas1-cas2*), and the *crispr* locus was located downstream of the *cas* gene clusters (Figure 2). In type II-C, the *crispr* locus was located downstream of the *cas* gene cluster *(cas9-cas1-cas2)* (Figure 2). Type III-A carried two *crispr* loci, including orphan *crispr1* and *crispr2* (Figure 2). The *crispr2* locus was adjacent to the *cas* gene clusters and was located downstream of the *cas* gene clusters. The orphan *crispr1* was located upstream of the *cas* gene clusters. Interestingly, some strains exhibited unique properties. A few strains encoded incomplete CRISPR-Cas systems. The strain NCTC12699 lacked the adaptation module (cas1-cas2) (Figure 2). The strain M1C152_1 had no Cas1 in the I-C system, while it showed integrity of the adaptation module in the II-C system (Figure 2). The strain NCTC11873 carried a III-A system with only one CRISPR locus (Table S2). In addition, we found that the orphan *crispr1* was located downstream of the *cas* gene clusters of the strain M1C142_1 (Figure 2).

###  Characterization of the repeat sequences and *cas1* genes 

**Figure 3 -:**
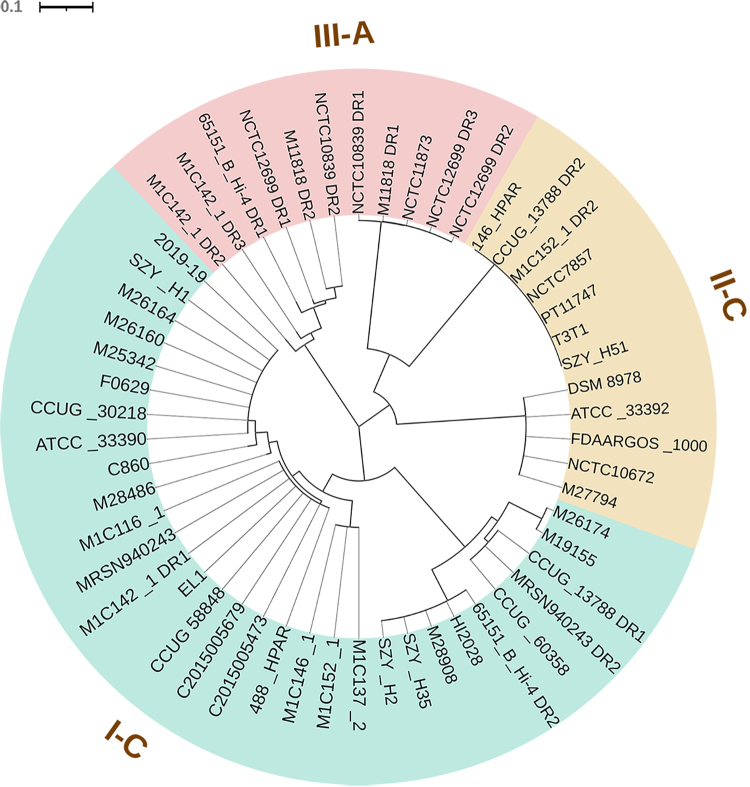
Phylogenetic tree of repeat sequences. Different regions are coloured by subtypes.

As a vital part of the CRISPR-Cas system, the repeat sequence plays a crucial role in the recognition and binding of Cas proteins (Wu et al., 2022). Therefore, repeat sequences were characterized. Overall, repeat sequences were highly conserved within the same subtype. The repeat sequences of subtype I-C consisted of 32 nucleotides (Figure 5a). The size of repeat sequences was 36 for subtype II-C, which was 37 for subtype III-A (Figure 5a). The stem-loop structure occurred in all repeat sequences. The identical secondary structure was found in all strains carrying the subtype I-C system (Figure 5a). Two different secondary structures were found in subtypes II-C and III-A (Figure 5c). Based on phylogenetic analysis, repeated sequences of the same subtype tended to cluster together (Figure 3). Interestingly, some repeats of subtype III-A clustered together with those of subtype II-C (Figure 3).

**Figure 4 -:**
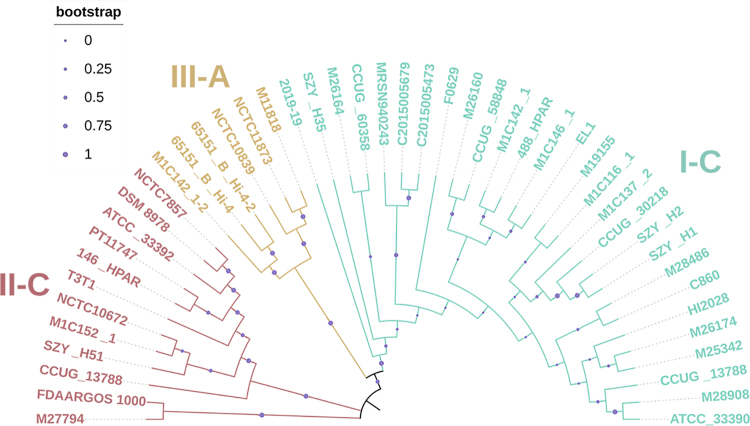
Phylogenetic tree of Cas1 sequences. Different regions are coloured by subtypes. Bootstrap values are represented by the size of the purple dots.

Cas1 protein usually serves as an indicator to reflect the evolutionary relationships of different systems due to its presence in almost all CRISPR-Cas systems (Makarova et al., 2011). Therefore, the phylogenetic tree based on *cas1* genes was created. As shown in Figure 4, all *cas1* genes were categorized into four distinct clades. However, the *cas1* genes from subtype II-C were divided into two main subclusters.

**Figure 5 -:**
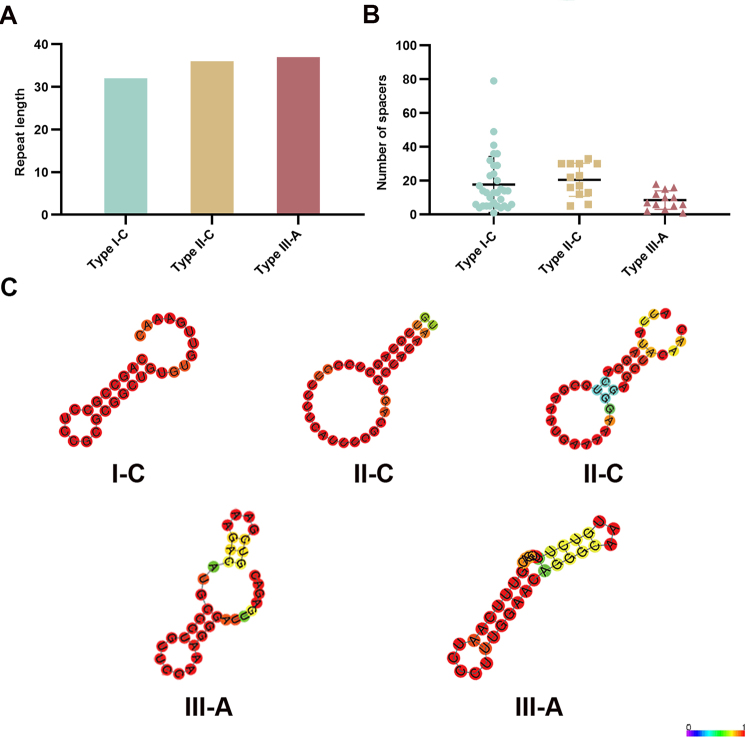
Characteristics of CRISPR-Cas systems in *Haemophilus* strains. (a) Size of repeat sequences within different Subtypes. (b) Number of spacers within different Subtypes. (c) Visualization of RNA secondary structure. Base-pair probability is indicated with different colors.

### Analysis of spacers and PAM prediction

Spacers act as “memory” and “recognition” in the CRISPR-Cas system. In total, 925 spacers were identified in 44 CRISPR-positive strains. As shown in Figure 5b, the type with the highest number of spacers was subtype II-C (n = 25), followed by subtypes I-C (n = 18) and III-A (n = 9). The strain M26174 contained the highest number of spacers, which carried 79 spacers. The strain carrying the least number of spacers was NCTC12699, and it contained 6 spacers (Table S2).

**Figure 6 -:**
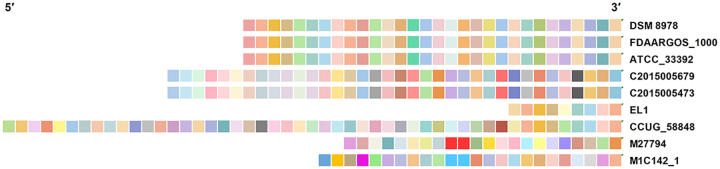
Visualization of spacers from representative CRISPR loci. Each distinct spacer is depicted as a rectangle with a unique colour. The most recent spacer is added at the right end, while the ancestral spacer is added at the left end.

The arrangement of spacers is typically considered to reflect the invasion sequence of exogenous DNA. In the CRISPR-Cas system, recent spacers are integrated at the leader end, and older spacers are added at the distal end (Barrangou et al., 2007). Accordingly, the arrangement of CRISPR spacers in *Haemophilus* strains was analysed and representative strains were selected to visualize the arrangements of spacers. The same arrangement of spacers was observed in different strains, such as FDAARGOS_1000, DSM 8978 and ATCC_33392 (Figure 6). Moreover, the identical arrangement of spacers was discovered in C2015005679 and C2015005473 as well (Figure 6). EL1 shared a similar arrangement from first to fifth with CCUG_58848 (Figure 6). Interestingly, two consecutive repeat spacers were discovered in the same strain, such as M27794 and M1C142_1 (Figure 6).

**Figure 7 -:**
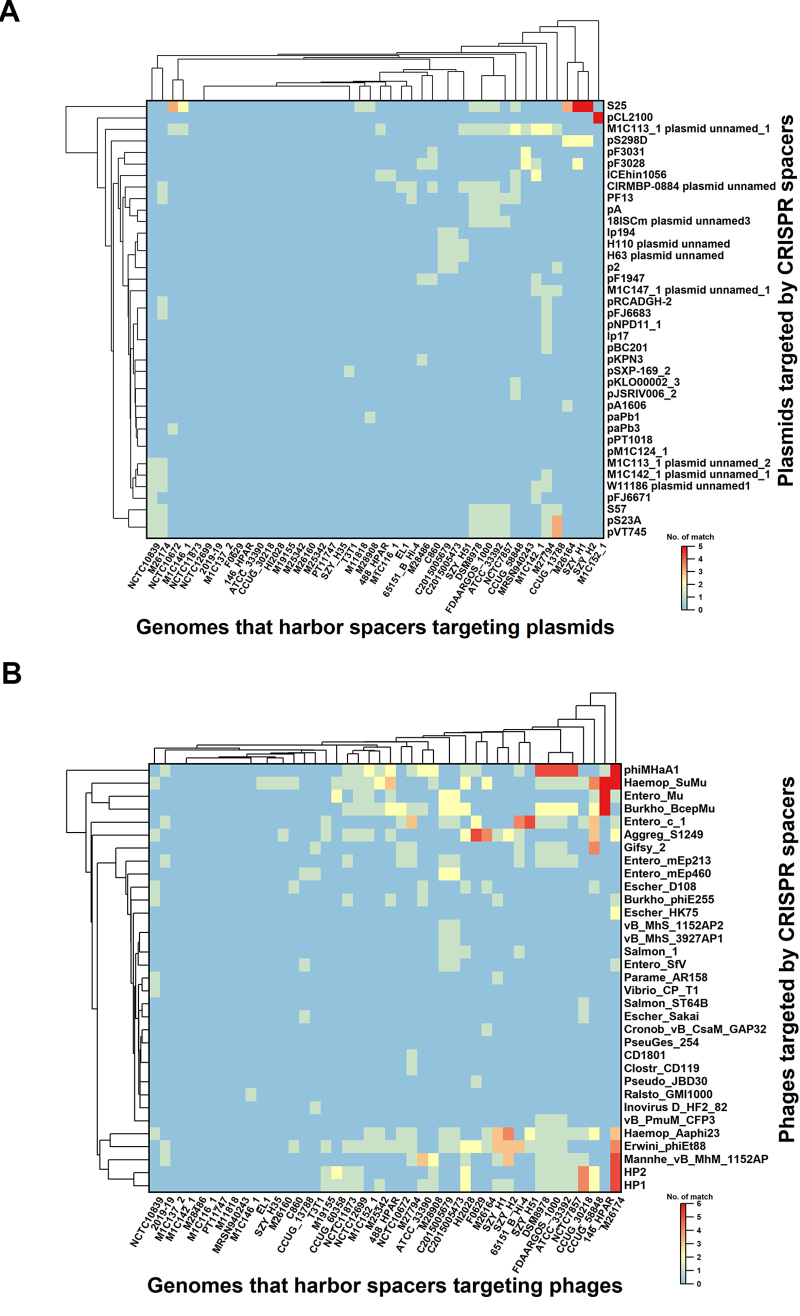
Plasmids (a) and phages (b) targeted by spacers in *Haemophilus* strains. The different colours were used to display frequency of targeted events.

Protospacers are foreign DNA fragments that are retained as “spacers” within the CRISPR arrays. To gain further insights into the origin of the spacer, the protospacers were identified by the CRISPRTarget server. The homology search showed that 29.62% (274/925) of the spacers were homologous to plasmids or phages (Figure 7). The spacers showed a strong targeting preference for phages (20.76%) rather than plasmids (8.86%) (Figure 7). The spacers from the strain M27794 matched the highest number of plasmids (Figure 7a). These spacers could target 12 different plasmids. M1C113_1 plasmid unnamed_1 was the most commonly targeted plasmid, and it was targeted 16 times by different strains (Figure 7a). In addition, we observed that the spacer from strain M26174 matched the highest number of phages (Figure 7b). The phiMHaA1 was the most frequent phage targeted by spacers, which was targeted 37 times (Figure 7b).

**Figure 8 -:**
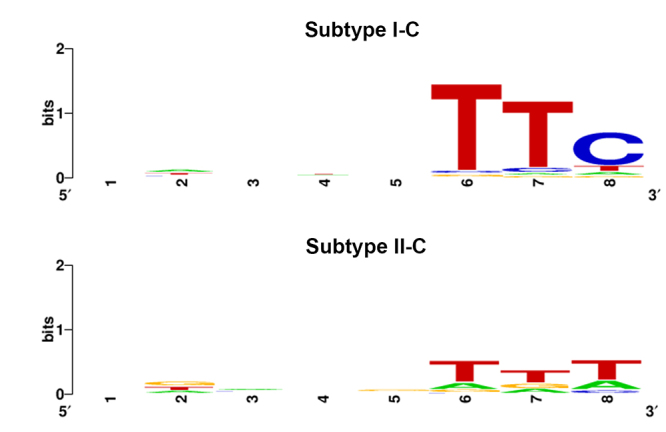
PAM prediction for subtype I-C and II-C systems in *Haemophilus* strains.

Understanding PAM sequences provides a reference for precise recognition and interference of target sequences. However, the location of PAM in the protospacers varies with the different subtypes. The PAM is present at the 5’ end of the protospacer for type I systems, while present at the 3’ end for type II systems (Vink et al., 2021). In this study, PAMs for subtype I-C were predicted to be 5’-TTC-3’ (Figure 8). The PAM for subtype II-C systems was identified as 5’-TTT-3’ (Figure 8). The PAM of subtype III-A remained unknown since the PAM location in subtype III-A was uncertain.

### Association of the CRISPR-Cas systems with virulence genes

The bacterial restriction system is an important factor influencing the acquisition and transfer of virulence factors. To analyze the relationship between CRISPR-Cas systems and virulence genes in *Haemophilus*, the occurrence of common virulence genes was investigated. *ompP5* (n=42, 30.4%) was the most prevalent virulence gene in *Haemophilus* strains, followed by *hmw2C* (n=23, 16.7%), *hmw1C* (n=23, 16.7%), *iga* (n=22, 15.9%), *hap* (n=20, 14.5%) (Table 1). Comparative analysis showed that CRISPR-positive strains carried more *ompP2* than CRISPR-negative strains (*P*=0.018), but the distribution of *hmw2C* and *hmw1C* showed a completely opposite trend (*P* < 0.05) (Table 1). The virulence gene *ompP2* was significantly more frequent in strains harboring type I-C CRISPR-Cas systems (*P*=0.027) (Table 1). A significant positive correlation was found between the virulence gene *hap* and the absence of the I-C subtype CRISPR-Cas system (*P*=0.007) (Table 1).

**Table 1 -:** The relationship between the CRISPR-Cas systems and virulence genes.

Genes	CRISPR-Cas system			Subtype I-C CRISPR-Cas system		
	Presence	Absence	*P* value	Presence	Absence	*P* value
	(N=44)	(N=94)		(N=31)	(N=107)	
*hmw2B*	0(0)	7(7.4%)	0.097	0(0)	7(6.5%)	0.349
*hia*	5(11.4%)	8(8.5%)	0.775	5(16.1%)	8(7.5%)	0.167
*ompP2*	8(18.2%)	4(4.3%)	0.018	6(19.4%)	6(5.6%)	0.027
*hifC*	0(0)	7(7.4%)	0.097	0(0)	7(6.5%)	0.349
*hmw1A*	0(0)	1(1.1%)	1.000	0(0)	1(0.9%)	1.000
*hifB*	0(0)	5(5.3%)	0.177	0(0)	5(4.7%)	0.587
*bexC*	0(0)	2(2.1%)	1.000	0(0)	2(1.9%)	1.000
*bexA*	0(0)	1(1.1%)	1.000	0(0)	1(0.9%)	1.000
*hmw2C*	3(6.8%)	20(21.3%)	0.048	2(6.5%)	21(19.6%)	0.104
*ompP5*	9(20.5%)	33(35.1%)	0.112	6(19.4%)	36(33.6%)	0.183
*hmw1C*	2(4.5%)	21(22.3%)	0.007	2(6.5%)	21(19.6%)	0.104
*hsf*	7(15.9%)	9(9.6%)	0.392	7(22.6%)	9(8.4%)	0.051
*hmw2A*	0(0)	2(2.1%)	1.000	0(0)	2(1.9%)	1.000
*hifA*	0(0)	5(5.3%)	0.177	0(0)	5(4.7%)	0.587
*bexD*	0(0)	2(2.1%)	1.000	0(0)	2(1.9%)	1.000
*hmw1B*	0(0)	7(7.4%)	0.097	0(0)	7(6.5%)	0.349
*hifD*	0(0)	5(5.3%)	0.177	0(0)	5(4.7%)	0.587
*iga*	4(9.1%)	18(19.1%)	0.211	2(6.5%)	20(18.7%)	0.161
*hifE*	0(0)	5(5.3%)	0.177	0(0)	5(4.7%)	0.587
*hap*	4(9.1%)	16(17.0%)	0.301	0(0)	20(18.7%)	0.007

Bold numers indicate statistically significant differences (*P*< 0.05).

## Discussion

The CRISPR-Cas system is a significant adaptive defense mechanism for prokaryotes against exogenous elements. MGEs are an important way to spread bacterial virulence. The role of CRISPR-Cas in the transmission of virulence differs across various bacteria. In this study, we analyzed the prevalence and characteristics of the CRISPR-Cas systems of *Haemophilus* spp. and explored their relationship with virulence genes.

Overall, CRISPR-Cas systems were detected in 44 out of 138 *Haemophilus* genomes, accounting for 31.88% of the total. The rate was significantly lower than the 40% occurrence rate assessed across bacteria (Burstein et al., 2016). The CRISPR-Cas system in *Haemophilus* included subtypes I-C, II-C and III-A. Subtype I-C was the most common system. The coexistence of two different subtypes could be observed in the same strains, such as strain 65151_B_Hi-4, M1C142_1, M1C152_1 and CCUG_13788. Similar phenomena were observed in other research, indicating CRISPR-Cas systems continue to compensate and enhance the defense ability as mobile elements evolved (Pinilla-Redondo et al., 2020). Specifically, when a system was unable to defend against MGE due to the absence of a certain element, such as the adaptation module, the element could be provided by another coexisting system to complete the effective protection. These findings explained that M1C152_1 lacked Cas1 in subtype I-C but had a complete adaptation module in subtype II-C. Cas1 and Cas2 proteins were essential for the initiation of the adaptation phase for the subtype III-A system (Zhang et al., 2022). The strain NCTC12699 exhibited the absence of *cas1* and *cas2* genes, which indicated that the strain NCTC12699 had lost the ability to obtain new interval sequences.

The highly conserved repeat sequences in the CRISPR-Cas system are crucial for its proper function. Repeat sequences of *Haemophilus* spp. were highly conserved within the same subtype. Interestingly, there were two different secondary structures in the subtype II-C. Phylogenetic analysis showed that some repeat sequences of subtype II-C clustered together with those of subtype III-A. The phylogenetic tree based on *cas1* genes showed different clusters of subtype II-C. These results suggested that the subtype II-C system in *Haemophilus* spp. had different evolution pathways.

Spacers serve as memories to reflect the spatial-temporal order of immune events (Mohanraju et al., 2016). The same arrangements of spacers were observed in different strains, indicating the close evolutionary relationship of these strains. EL1 shared a similar arrangement from first to fifth with CCUG_58848, indicating that they might originate from the same ancestor or at least appeared in the same environment at a certain point. Additionally, the strain M27794 contained two identical spacer sequences, both targeting the phages. We speculate that this may be due to the phage-bacteria arms race (Hampton et al., 2020). On the one hand, phages could overcome type I and type II systems due to mutations in the PAMs and the protospacers. However, there was a positive feedback mechanism in type I and type II systems. The mechanism helped CRISPR-Cas systems boost immunity by adding multiple new spacers against escaped phages. On the other hand, some phages could produce anti-CRISPR (Acr) proteins. These Acr proteins blocked the activity of Cas proteins. Acr proteins exhibited transient immunosuppression but might fail to completely protect subsequent phages from the CRISPR-Cas systems. Understanding the origin of spacers will provide insights into the mechanisms of interaction between CRISPR-Cas systems and MGEs. The result showed that 29.62% (274/925) of the spacers showed significant matches to a plasmid or phage, close to the 32% match rate estimated in previous studies (Vink et al., 2021). The limited match rate was attributed to multiple reasons, including undeveloped MGE sequences and self-targeting spacers (Stern et al., 2010). In addition, the match rate of spacers to plasmids (82/925, 8.86%) was significantly lower than the match rate to phages (192/925, 20.76%).

Virulence is a key factor in triggering bacterial infections. In this study, some virulence genes were negatively correlated with the CRISPR-Cas system, such as *hmw2C* and *hmw1C*. However, the virulence gene *ompP2* shows a significantly positive correlation with type I-C CRISPR-Cas systems. This suggested that the CRISPR-Cas system may play a dual role in the virulence of *Haemophilus*. There were two potential mechanisms: (i) the defense of CRISPR-Cas systems may diminish potential virulence by inhibiting the invasion of exogenous DNA with virulence genes; (ii) CRISPR-Cas systems may enhance bacterial virulence by controlling gene expression (Wu et al., 2022; Paauw et al., 2025).

In conclusion, the study analyzed the characteristics of the CRISPR-Cas system and its association with virulence in the genus *Haemophilus*. The results indicate that the CRISPR-Cas systems in *Haemophilus* spp. exhibit strong subtype dependence. Further findings suggest that the CRISPR-Cas systems may play a dual role in *Haemophilus* virulence. However, due to the limited number of genomic samples, the findings regarding the prevalence of CRISPR-Cas systems and their association with virulence genes remain preliminary. The generalizability of these findings requires further validation with larger genomic datasets. Nonetheless, this study enhances our understanding of the structural features, functional diversity, and evolutionary trends of CRISPR-Cas systems in *Haemophilus* genus, and provides a theoretical basis for further investigation into their relationship with virulence factors.

## Supplementary material

The following online material is available for this article:

Table S1 - Genomic information of analyzed *Haemophilus* in this study.

Table S2 - Identified CRISPR/Cas systems in *Haemophilus* strains.

## Data Availability

 The entire dataset supporting the results of this study was published in the article and in the Supplementary Material section.
