# Supplementary material for: Characterization of CRISPR-Cas systems in the Haemophilus genus CRISPR-Cas in Haemophilus spp
Source: Genet Mol Biol. 2026 Mar 16;49(1):e20250166. doi: 10.1590/1678-4685-GMB-2025-0166 (PMC13016550; doi:10.1590/1678-4685-GMB-2025-0166)
Supplement: Table S1 - [file 1415-4757-GMB-49-01-e20250166-s1.pdf]

Genetics and Molecular Biology
https://doi.org/10.1590/1678-4685-GMB-2025-0166

Supplementary Material to "Characterization of CRISPR-Cas systems in the *Haemophilus* genus CRISPR-Cas in *Haemophilus* spp."

Table S1 - Genomic information of analyzed *Haemophilus* in this study.

Strains	Genome Size (Mbp)	GC (%)	Assembly Level	Accession number
FDAARGOS_1199	2.093	40.5	Complete	SAMN16357341
NCTC13334	2.181	42.5	Complete	SAMEA4384241
NCTC8502	1.993	38.5	Complete	SAMEA4076725
477	1.846	38.0	Complete	SAMN02595602
VAN2	1.590	38.0	Complete	SAMN04632440
NCTC10839	1.935	38.5	Complete	SAMEA3905387
FDAARGOS_1560	1.891	38.0	Complete	SAMN22091657
NCTC8143	1.891	38.0	Complete	SAMEA2498480
FDAARGOS_1000	2.142	39.5	Complete	SAMN16357169
DSM 8978	2.142	39.5	Complete	SAMN37162770
5P54H1	1.886	38.0	Complete	SAMN06203639
CHBN-III-6	1.885	38.0	Complete	SAMD00119538
CHBN-IV-1	1.883	38.0	Complete	SAMD00119541
NCTC12699	1.880	38.0	Complete	SAMEA3221100
M1C137_2	2.177	39.5	Complete	SAMN16409249
EL1	2.153	39.5	Complete	SAMN34140652
M27794	2.149	39.5	Complete	SAMN09760371
LC_1315_18	2.068	39.5	Complete	SAMN10774168
NCTC10665	2.062	39.5	Complete	SAMEA104062569
M1C113_1	2.089	39.5	Complete	SAMN16409224
M1C152_1	2.023	39.5	Complete	SAMN16409277
M1C160_1	2.018	39.5	Complete	SAMN16409282
M1C149_1	2.015	39.5	Complete	SAMN16409271
M1C142_1	2.038	39.5	Complete	SAMN16409259
M1C120_2	2.001	39.5	Complete	SAMN16409231
M1C130_2	1.995	39.5	Complete	SAMN16409239
M1C125_4	1.953	39.5	Complete	SAMN16409233
M1C147_1	1.982	39.5	Complete	SAMN16409268
M1C146_1	1.927	39.5	Complete	SAMN16409265
T3T1	2.087	39.5	Complete	SAMEA3138384
GHA9	1.776	38.0	Complete	SAMN04632449

Strains	Genome Size (Mbp)	GC (%)	Assembly Level	Accession number
GHA8	1.770	38.0	Complete	SAMN04632448
GHA5	1.739	38.0	Complete	SAMN04632447
GHA3	1.739	38.0	Complete	SAMN04632446
FDAARGOS_297	1.701	38.0	Complete	SAMN06173310
VAN4	1.673	38.0	Complete	SAMN04632442
VAN5	1.667	38.0	Complete	SAMN04632443
VAN1	1.667	38.0	Complete	SAMN04632439
VAN3	1.667	38.0	Complete	SAMN04632441
GHA2	1.634	38.0	Complete	SAMN04632445
GHA1	1.622	38.0	Complete	SAMN04632444
NCTC11483	1.594	38.0	Complete	SAMEA4412686
35000HP	1.699	38.0	Complete	SAMN02604087
M19346	1.973	38.5	Complete	SAMN09704955
M19345	1.916	38.5	Complete	SAMN09704954
2019-19	1.895	38.5	Complete	SAMD00251501
M28486	1.823	38.5	Complete	SAMN09704968
NEB129	2.092	40.5	Complete	SAMN11345835
NCTC8134	1.993	38.5	Complete	SAMEA26395168
F0629	1.806	38.5	Complete	SAMN08439034
SZY H68	1.903	38.5	Complete	SAMN25235948
93P12H1	1.812	38.0	Complete	SAMN31697148
ATCC 33392	2.125	39.0	Scaffold	SAMN00253308
CCUG 43573	2.047	45.5	Scaffold	SAMN06308712
CCUG 4438	1.535	37.5	Scaffold	SAMN06308731
NCTC 7857	2.148	39.5	Contig	SAMEA53433418
ATCC 33940	1.577	38.0	Contig	SAMN02983000
ATCC 33390	1.828	38.5	Contig	SAMN03217754
CCUG 12834	1.827	38.5	Scaffold	SAMN05043237
SZY H1	2.062	38.0	Scaffold	SAMN11775792
CCUG 12834	1.827	38.5	Scaffold	SAMN05043237
NCTC 8479	2.096	40.5	Contig	SAMEA87363418
CCUG 13788	2.144	39.5	Contig	SAMN02393707
HK385	2.031	40.0	Contig	SAMN00761805
HK 85	2.183	42.5	Contig	SAMN00621708
CCUG 3718	2.058	41.0	Scaffold	SAMN06308708
NCTC10672	2.236	39.5	Contig	SAMEA3207621
209_HPAR	1.997	39.5	Scaffold	SAMN03197401
CCUG 58848	2.148	39.0	Scaffold	SAMN05219234
M1C111_2	1.969	39.5	Contig	SAMN16409219
146_HPAR	2.029	39.5	Scaffold	SAMN03197335
M1C116_1	1.974	39.5	Contig	SAMN16409227

Strains	Genome Size (Mbp)	GC (%)	Assembly Level	Accession number
488_HPAR	2.112	39.5	Contig	SAMN03197680
M19066	1.845	38.5	Contig	SAMN09704940
65151 B Hi-4	1.898	38.0	Contig	SAMN10537231
M26160	1.911	38.5	Contig	SAMN09704960
M26157	1.873	38.5	Contig	SAMN09704959
M11818	1.962	38.5	Contig	SAMN09704939
M19080	1.945	38.5	Contig	SAMN09704943
M26164	1.812	38.5	Contig	SAMN09704962
M25342	1.866	38.5	Contig	SAMN09704957
M28908	1.758	38.5	Contig	SAMN09704969
M26174	1.975	39.0	Contig	SAMN09704966
M19155	1.905	38.5	Contig	SAMN09704948
M19135	1.947	39.0	Contig	SAMN09704946
HI2028	1.849	38.5	Contig	SAMN03217745
65117 B Hi-3	1.929	38.0	Contig	SAMN10537228
M19122	1.979	38.5	Contig	SAMN09704945
16-549009	1.843	38.5	Scaffold	SAMN10405313
CCUG 30218	1.987	38.0	Contig	SAMN10537237
CCUG 11096	1.934	38.0	Contig	SAMN10537235
UMB0862	2.167	39.0	Contig	SAMN34996463
C2015005679	2.002	39.5	Contig	SAMN09011121
C2002001239	2.159	39.5	Contig	SAMN09011141
C2015005473	2.000	39.5	Contig	SAMN09011122
MRSN940243	2.024	39.5	Contig	SAMN29768393
C860	2.123	38.5	Contig	SAMN05570529
SZY H8	1.856	38.0	Contig	SAMN19323121
SZY H35	1.916	38.0	Contig	SAMN19365525
SZY H68	1.866	38.0	Contig	SAMN19365527
SZY H51	2.156	39.5	Contig	SAMN34146969
CCUG 60358	2.091	39.5	Scaffold	SAMN05225442
HMSC71H05	2.025	39.5	Scaffold	SAMN04498897
65001_BAL_Hi1	1.951	38.0	Scaffold	SAMN09845281
M08964	1.803	38.0	Contig	SAMN09704874
65001_NP_Hi2	1.948	38.0	Contig	SAMN09845280
60295_BAL_Hi1	1.816	38.0	Scaffold	SAMN09845269
M10910	1.931	38.0	Contig	SAMN09704886
PT12088	1.808	38.0	Contig	SAMN27398407
PT11400	1.808	38.0	Contig	SAMN27398394
PT10821	1.808	38.0	Contig	SAMN27398371
PT11098	1.809	38.0	Contig	SAMN27398388
PT10923	1.808	38.0	Contig	SAMN27398372

Strains	Genome Size (Mbp)	GC (%)	Assembly Level	Accession number
PTHi-10167	1.850	38.0	Contig	SAMEA4643466
M24275	1.936	38.0	Contig	SAMN09704923
2842STDY5882019	1.836	38.0	Scaffold	SAMEA2674679
2842STDY5882040	1.840	38.0	Scaffold	SAMEA2674722
2842STDY5882018	1.836	38.0	Scaffold	SAMEA2674677
HI1426	1.847	38.0	Contig	SAMN03702704
GE42	1.788	38.0	Contig	SAMN04590164
M15927	1.863	38.0	Contig	SAMN09704906
Hi805	1.777	38.0	Contig	SAMN31888532
M17527	1.863	38.0	Contig	SAMN09704910
M3835	1.797	38.0	Contig	SAMN09704937
M22533	1.831	38.0	Contig	SAMN09704919
PTHi-13476	1.832	38.0	Contig	SAMEA4643507
2842STDY5882047	1.828	38.0	Scaffold	SAMEA2674736
M21328	1.791	38.0	Contig	SAMN09704914
PTHi-4419	1.893	38.0	Contig	SAMEA4643448
M22154	1.810	38.0	Contig	SAMN09704918
H	1.867	38.0	Contig	SAMN12390026
M24280	1.899	38.0	Contig	SAMN09704924
P647-8506	1.860	38.0	Contig	SAMN07421924
M14791	1.902	38.0	Contig	SAMN09704903
P675-1755	1.760	38.0	Contig	SAMN07421950
PTHi-11358	1.781	38.0	Contig	SAMEA4643473
M17128	1.847	38.0	Contig	SAMN09704909
SZY_H2	2.059	38.0	Scaffold	SAMN12740661
