# Supplementary material for: Characterization of CRISPR-Cas systems in the Haemophilus genus CRISPR-Cas in Haemophilus spp
Source: Genet Mol Biol. 2026 Mar 16;49(1):e20250166. doi: 10.1590/1678-4685-GMB-2025-0166 (PMC13016550; doi:10.1590/1678-4685-GMB-2025-0166)
Supplement: Table S2 - [file 1415-4757-GMB-49-01-e20250166-s2.pdf]

Genetics and Molecular Biology
https://doi.org/10.1590/1678-4685-GMB-2025-0166

Supplementary Material to "Characterization of CRISPR-Cas systems in the *Haemophilus* genus CRISPR- Cas in *Haemophilus* spp."

Table S2 - Identified CRISPR/Cas systems in *Haemophilus* strains.

Species	Strains	System Type	Repeat sequences	Repeat length	No. spacers
H. haemolyticus	NCTC10839	III-A	GTTTCAATCCCTTTGGAACAGGGCAATGTCTTTCGAC	37	15
		III-A	GTCGAAAGACATGCCCTGTTCCAAAGGGATTGAGAC	36	12
	2019-19	I-C	CAGCCGCCTCCGCGCGGCTGTGTGTTGAAAC	31	4
	M28486	I-C	GCAGCCGCCTTCGCGCGGCTGTGTGTTGAAAC	32	6
	M19066	I-C	GTTTCAACACACAGCCGCACGAAGGCGGCTGC	32	29
	ATCC_33390	I-C	GCAGCCGCCTCCGCGCGGCTGTGTGTTGAAAC	32	17
	65151_B_Hi-4	III-A	GTCTCAATCCCTTTGAAACAGGGCAATGTCTTTCGAC	37	10
		III-A	GTCTCAATCCCTTTGAAACAGGGCAATGTCTTTCGAC	37	6
		III-A	GTCGAAAGACATTGCCCTGTTCCAAAGGGATTGA	34	5
		I-C	GTTTCAACACACAGCCGCGCGGAGGCGGCTGC	32	13
	M26160	I-C	GCAGCCGCCTCCGCGCGGCTGTGTGTTGAAAC	32	7

Species	Strains	System Type	Repeat sequences	Repeat length	No. spacers
	M11818	III-A	GTTTCAATCCCTTTGGAACAGGGCAATGTCTTTCGAC	37	10
		III-A	GTCGAAAGACATTGCCCTGTTCCAAAGGGATTGAGAC	37	18
	M26164	I-C	GCAGCCGCCTCCGCGCGGCTGTGTGTTGAAAC	32	24
	M25342	I-C	GCAGCCGCCTCCGCGCGGCTGTGTGTTGAAAC	32	5
	M28908	I-C	GTTTCAACACACAGCCGCGCGGAGGCGGCTGC	32	29
	M26174	I-C	GTTTCAACACACAGCCACCCGAAGGTGGCTGC	32	79
	HI2028	I-C	GTTTCAACACACAGCCGCGCGGAGGCGGCTGC	32	14
	M19155	I-C	GTTTCAACACACAGCCACCCGAAGGTGGCTGC	32	6
	CCUG_30218	I-C	GCAGCCGCCTCCGCGCGGCTGTGTGTTGAAAC	32	14
H. parainfluenze	FDAARGOS_1000	II-C	GTTGTAGCTCCCTTTTTCATTTCGCAGTGCTATAAT	36	30
	DSM 8978	II-C	GTTGTAGCTCCCTTTTTCATTTCGCAGTGCTATAAT	36	30
	M1C137_2	I-C	GCAGCCACCTTCGGGTGGCTGTGTGTTGAAAC	32	4
	EL1	I-C	GCAGCCGCCTTCGGGCGGCTGTGTGTTGAAAC	32	9
	M27794	II-C	GTTGTAGCTCCCTTTTTCATTTCGCAGTGCTATAAT	36	22
	M1C152_1	I-C	GCAGCCACCTTCGGGTGGCTGTGTGTTGAAACT	33	5
		II-C	ATTATAGCACTGCGAAATGAAAAAGGGAGCTACAAC	36	17
	M1C142_1	I-C	GCAGCCGCCTTCGGGCGGCTGTGTGTTGAAAC	32	13
		III-A	GTCTCAATCCCTTTCGAGCAGGGCGATGTCTTTCGAC	37	6

Species	Strains	System Type	Repeat sequences	Repeat length	No. spacers
		III-A	GTCTCAATCCCTTTCGAACAGGGCTATGTCTTTCGAC	37	16
	M1C146_1	I-C	GCAGCCACCTTCGGGTGGCTGTGTGTTGAAAC	32	10
	T3T1	II-C	ATTATAGCACTGCGAAATGAAAAAGGGAGCTACAAC	36	16
	ATCC_33392	II-C	GTTGTAGCTCCCTTTTTCATTTCGCAGTGCTATAAT	36	30
	NCTC7857	II-C	ATTATAGCACTGCGAAATGAAAAAGGGAGCTACAAC	36	30
	NCTC10672	II-C	GTTGTAGCTCCCTTTTTCATTTCGCAGTGCTATAAT	36	33
	CCUG_58848	I-C	GCAGCCGCCTTCGGGCGGCTGTGTGTTGAAAC	32	49
	M1C116_1	I-C	GCAGCCGCCTTCGGGCGGCTGTGTGTTGAAAC	32	5
	146_HPAR	II-C	ATTATAGCACTGCGAAATGAAAAAGGGAGCTACAAC	36	6
	488_HPAR	II-C	ATTATAGCACTGCGAAATGAAAAAGGGAGCTACAAC	36	12
		I-C	GCAGCCGCCTTCGGGCGGCTGTGTGTTGAAAC	32	41
H. influenzae	NCTC11873	III-A	GTCGAAAGACATTGCCCTGTTCCAAAGGGATTGAGAC	37	7
	NCTC12699	III-A	GTCTCAATCCCTTTGGAACAGGGCAATGTCTTTCGAC	37	2
		III-A	GTCGAAAGACATTGCCCTGTTCCAAAGGGATTGAGAC	37	3
		III-A	GTCGAAAGACATTGCCCTGTTCCAAAGGGATTGAGAC	37	1
H. sp. oral taxon 036	F0629	I-C	GCAGCCGCCTCCGCGCGGCTGTGTGTTGAAAC	32	23
H.seminalis	SZY_H2	I-C	GTTTCAACACACAGCCGCGCGGAGGCGGCTGC	32	32
	SZY_H1	I-C	GCAGCCGCCTCCGCGCGGCTGTGTGTTGAAAC	32	20

Species	Strains	System Type	Repeat sequences	Repeat length	No. spacers
	CCUG_13788	I-C	GTTTCAACACACAGCCGCCCGAAGGCGGCTGC	32	13
		II-C	ATTATAGCACTGCGAAATGAAAAAGGGAGCTACAAC	36	13
H.sputorum	C2015005679	I-C	GCAGCCGCCTTCGGGCGGCTGTGTGTTGAAAC	32	36
	MRSN940243	I-C	GTTTCAACACACAGCCGCCCGAAGGCGGCTGC	32	15
		I-C	GCAGCCGCCTTCGGGCGGCTGTGTGTTGAAAC	32	5
	C2015005473	I-C	GCAGCCGCCTTCGGGCGGCTGTGTGTTGAAAC	32	36
Unclassified Haemophilus	SZY_H35	I-C	GTTTCAACACACAGCCGCGCGGAGGCGGCTGC	32	4
	SZY_H51	II-C	ATTATAGCACTGCGAAATAAAAAAGGGAGCTACAAC	36	5
	CCUG_60358	I-C	GTTTCAACACACAGCCGCCCGAAGGCGGCTGC	32	14
	C860	I-C	GCAGCCGCCTCCGAGCGGCTGTGTGTTGAAAC	32	5
